# Porous silicon nanoparticles in drug delivery, regenerative medicine, and biosensing: a translational review

**DOI:** 10.3389/fbioe.2026.1790476

**Published:** 2026-09-14

**Authors:** Yin Ding, Bin Ma

**Affiliations:** 1 Key Lab of Stomatology of State Ethnic Affairs Commission, Northwest Minzu University, Lanzhou, Gansu, China; 2 Key Lab of Oral Diseases of Gansu Province, Northwest Minzu University, Lanzhou, Gansu, China

**Keywords:** porous silicon nanoparticles, drug delivery, tissue regeneration, biosensing, clinical translation, surface functionalization

## Abstract

Porous silicon nanoparticles (pSiNPs) have become a versatile platform for biomedical applications owing to their tunable pore structure, high surface area (200–800 m^2^/g), intrinsic biodegradability into orthosilicic acid, and versatile surface chemistry. This review systematically examines the synthesis strategies (electrochemical etching, magnesiothermic reduction, stain etching, laser ablation), composite fabrication with biocompatible polymers (PCL, PLGA, chitosan, PEG), and surface functionalization approaches that govern pSiNP performance in biological environments. We evaluate preclinical evidence across four major application domains: (i) targeted drug delivery, where pH-responsive and MMP-cleavable gating strategies have demonstrated up to 7-fold lower off-target toxicity in murine tumor models; (ii) regenerative medicine, including non-load-bearing bone repair (2.1–2.3-fold increase in BV/TV in rodent calvarial defects) and chronic wound healing (95% wound closure by day 14 in diabetic mice); (iii) implant surface modification, with pSiNP coatings improving osseointegration and reducing capsular contracture in rabbit models; and (iv) biosensing and imaging, achieving fM–pM detection limits and tumor-to-background ratios >8:1. Despite these advances, all current evidence remains at the *in vitro* or preclinical stage; no pSiNP-based product has entered clinical trials. Critical barriers to translation include batch-to-batch manufacturing variability, unpredictable *in vivo* degradation kinetics, mechanical fragility under load-bearing conditions, and the absence of harmonized regulatory standards for combination products. We propose a three-pillar framework—predictive in vitro–in vivo correlation, modular surface engineering, and GMP-compliant manufacturing—as a roadmap toward rational clinical development.

## 1 Introduction

Repair of bone tissue defects and chronic wounds represents a significant clinical challenge. Traditional treatment approaches are often limited by donor shortages, immune rejection, and suboptimal repair efficiency, necessitating the development of novel biomaterials with excellent bioactivity, controllable degradation properties, and superior mechanical performance. Silicon-based materials have garnered considerable attention in the field of regenerative medicine in recent years due to their unique ion release capacity, promotion of angiogenesis and osteogenic differentiation, and superior biocompatibility.

**FIGURE 1:**
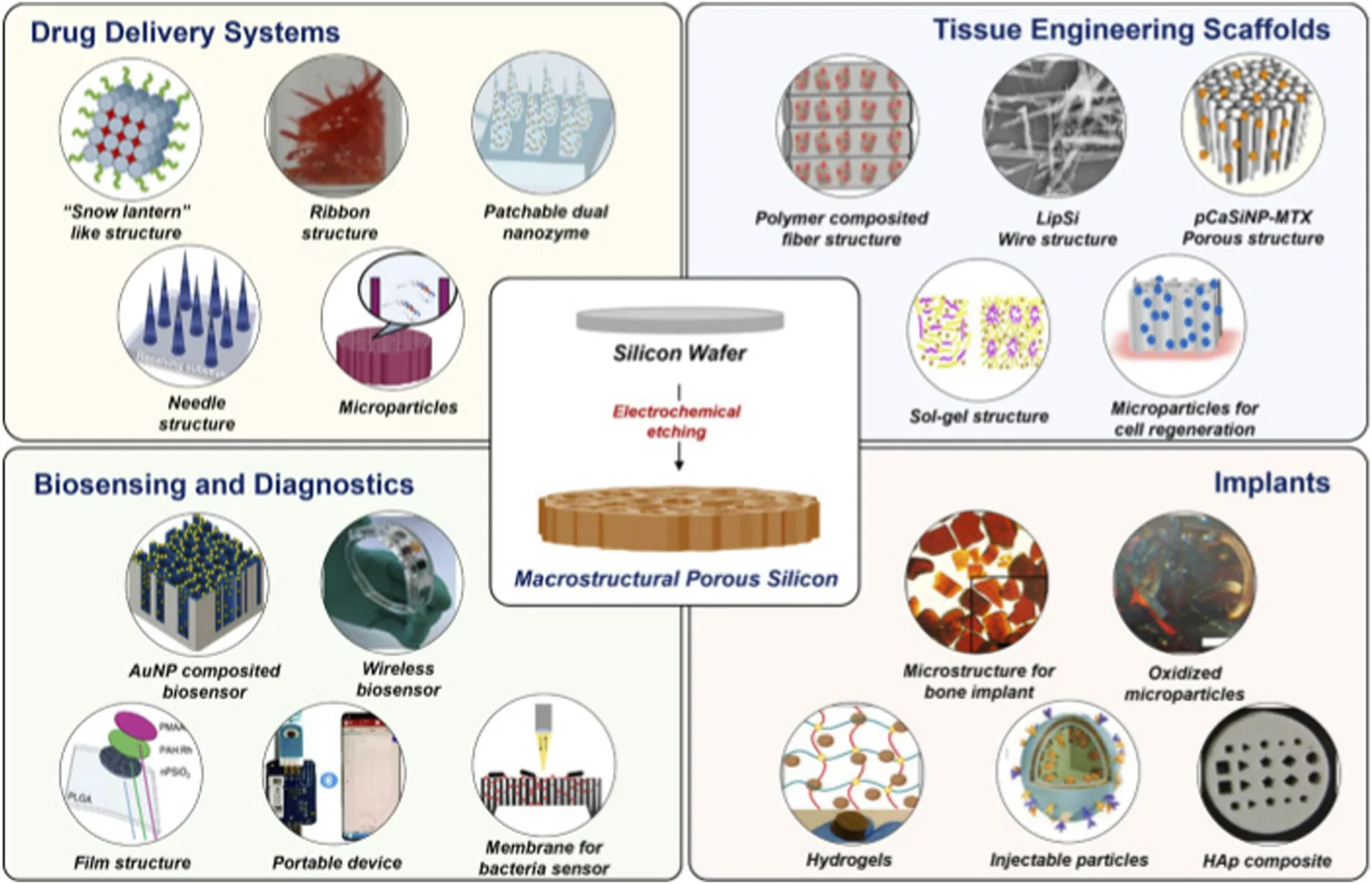
Biomedical applications of macrostructural porous silicon (Kang et al., 2025). Unique properties of porous silicon nanoparticles (pSiNPs) and their diverse clinical applications.

Over the past 15 years, porous silicon nanoparticles (pSiNPs) have emerged as a promising biodegradable platform in the biomedical field because of their distinctive combination of high surface area, tunable pore structure, intrinsic photoluminescence, and tunable degradation into biocompatible silicic acid (Park et al., 2019; Henstock et al., 2015). These unique properties make pSiNPs suitable for a variety of clinical applications, including targeted drug delivery, regenerative medicine, biosensing, and functionalization of medical devices. Significant progress has been made in pSiNP synthesis, surface design, and composite formation to improve performance and biocompatibility (Lee et al., 2018; Du et al., 2022; Lou et al., 2025). Despite these advances, translation of pSiNPs from bench to bedside remains impeded by challenges in manufacturing scalability, batch-to-batch homogeneity, *in vivo* degradation predictability, and regulatory alignment (Kumeria et al., 2017; Go et al., 2020) as shown in Figure 1.

Previous reviews have addressed individual aspects of pSiNP research, including drug delivery applications (Kumeria et al., 2017), biosensing, and biodegradation (Go et al., 2020). However, no existing review has systematically integrated the complete translational chain from synthesis through composite fabrication, surface modification, targeted delivery, regenerative repair, implant modification, biosensing, and clinical translation barriers into a single cohesive framework. The novelty of the present review lies in four aspects: (1) to our knowledge, it is the first to comprehensively map the entire translational pipeline of pSiNPs from bench to potential bedside applications; (2) it explicitly stratifies the level of evidence (*in vitro*, animal, clinical) for each application domain; (3) it provides a head-to-head comparison of pSiNPs with other silicon-based materials (solid SiNPs, mesoporous silica, Si_3_N_4_) to clarify niche advantages; and (4) it proposes a three-pillar clinical translation framework centered on predictive in vitro–in vivo correlation, modular surface engineering, and GMP-compliant continuous manufacturing. By addressing these gaps, this review provides a roadmap to accelerate pSiNPs from promising laboratory constructs toward rational, regulated clinical evaluation.

## 2 Review methodology

A structured literature search was conducted to identify relevant studies on porous silicon nanoparticles and related silicon-based biomaterials for biomedical applications.

### 2.1 Data sources

PubMed/MEDLINE, Web of Science Core Collection, and Scopus were searched from January 2014 to January 2026, with a focused emphasis on publications from the most recent 5 years (2021–2026).

### 2.2 Search terms

The following keyword combinations were employed (“porous silicon nanoparticles” OR “mesoporous silicon” OR “porous silicon” OR “silicon-based biomaterials”) AND (“drug delivery” OR “tissue regeneration” OR “bone repair” OR “wound healing” OR “biosensing” OR “bioimaging” OR “clinical translation” OR “biocompatibility” OR “degradation”).

### 2.3 Inclusion criteria

(i) Original research articles and peer-reviewed reviews reporting experimental data on pSiNPs or closely related silicon nanomaterials (mesoporous silica nanoparticles, silicon nanowires, oxidized porous silicon) in biomedical contexts; (ii) Studies describing synthesis, characterization, surface modification, drug loading/release, biological evaluation (*in vitro*, *ex vivo*, or *in vivo*), or translational/regulatory aspects; (iii) Articles published in English in peer-reviewed journals.

### 2.4 Exclusion criteria

(i) Studies focused exclusively on non-biomedical applications (e.g., lithium-ion battery anodes, photovoltaics, electronics); (ii) Non-peer-reviewed preprints, conference abstracts without full experimental data; (iii) Studies involving silicon materials without clear relevance to nanoparticle-based biomedical applications.

### 2.5 Study selection and data extraction

A total of 65 references were ultimately included, comprising original research articles, review articles, and relevant regulatory guidance documents. Key data extracted included synthesis parameters, surface modification strategies, application domains, experimental models (cell lines, animal models), key outcomes, and evidence level (*in vitro*, animal, clinical).

## 3 Preparation and composite modification methods of porous silicon nanoparticles

### 3.1 Porous silicon nanoparticle synthesis techniques

Since the early 1990s, porous silicon nanoparticles (pSiNPs) have been developed as a group of nanomaterials with excellent tunable physicochemical properties, biocompatibility, and biodegradability, supporting diverse applications in drug delivery, regenerative medicine, and biosensing (Betty, 2008). The preparation of pSiNPs mainly relies on top-down methods to convert bulk crystalline silicon into nanostructured porous silicon.

Electrochemical etching is the most widely used approachA silicon wafer (typically doped with boron or phosphorus) is anodized in an HF electrolyte solution at a defined current density. The resulting pSi layer can be detached from the wafer by ultrasonic agitation or thermal shock, then size-reduced by ball milling and probe sonication to nanoparticles of 50–300 nm. Key parameters—including doping type, current density (10–100 mA/cm^2^), HF concentration, and etching duration—determine the pore morphology, porosity (typically 40%–80%), and particle size distribution (Lv et al., 2017).

Magnesiothermic reduction involves the reaction of SiO_2_ nanoparticles with magnesium vapor at high temperatures (650 °C–700 °C) to produce highly porous silicon while retaining the original spherical morphology, with tunable pore distribution (Aguilera-Correa et al., 2022). This method offers scalability advantages over electrochemical etching but may leave residual MgO impurities requiring additional purification steps. The influence of reaction parameters on product quality is notable: adjusting the Mg/SiO_2_ ratio and temperature within the 650 °C–700 °C window permits precise control over pore size distribution and surface area (Ma et al., 2023).

Stain etching is a simpler chemical route in which Si wafers are immersed in a mixture of HF and HNO_3_ to form pSiNPs without the need for electrical equipment. However, this method produces less uniform pore distributions than electrochemical routes (Aguilera-Correa et al., 2022).

Laser ablation in liquid has been recently exploited for the fabrication of crystalline pSiNPs from porous silicon substrates or silicon nanowires. This process yields particles <50 nm in diameter with strong optical photoemission in the visible-to-NIR range, suitable for optical bioimaging. The key advantage is that it avoids chemical reagents entirely, though yield remains low and equipment costs are high. A comprehensive review of laser-processed nanosilicon by Kabashin et al. established that this approach yields crystalline nanoparticles with tunable photoluminescence in the 650–850 nm range and favorable colloidal stability through controlled surface oxidation (Kabashin et al., 2019).

All synthesis routes share the same biomedical goals: high surface area (>200 m^2^/g), controlled degradation rate, and minimal toxicity. The precursor morphology plays a decisive role in determining final product characteristics—complete conversion of vertically aligned mesoporous silica channels via magnesiothermic reduction can yield pure mesoporous silicon nanoparticles while preserving the original hierarchical pore architecture (Kim et al., 2015). The synthesis conditions and subsequent aging of the particles also matter: photocatalytic activity of mesoporous silicon nanoparticles has been shown to depend significantly on both magnesiothermic reduction parameters and storage duration (Curtis et al., 2021) as shown in Table 1.

**TABLE 1:** Characteristics of key pSiNP synthesis techniques.

Method	Precursor/Conditions	Particle size (nm)	Porosity (%)	Key advantages	Limitations	Ref.
Electrochemical Etching	HF/EtOH, 10–100 mA/cm^2^	50–300	40–80	Tunable pore size, high reproducibility	Requires p/n-type Si wafer, conductive substrate, HF handling hazards	(Lv et al., 2017)
Magnesiothermic Reduction	SiO_2_ + Mg, 650 °C–700 °C, Ar atmosphere	80–250	50–75	Scalable, spherical morphology	High temperature, residual MgO impurities	(Aguilera-Correa et al., 2022)
Laser Ablation	Porous Si target, H_2_O/ethanol, pulsed laser	20–60	30–60	Crystalline phase, no chemicals	Low yield, expensive equipment	(Aguilera-Correa et al., 2022)
Stain Etching	Si wafer, HF/HNO_3_ (3:1), RT	100–400	45–70	Simple, no power supply	Irregular pores, broad size distribution	(Aguilera-Correa et al., 2022)

### 3.2 Composite strategies of pSiNPs with biocompatible polymers

Porous silicon has low mechanical strength and high brittleness, with compressive strength far below that of natural bone tissue. It cannot be directly used as a structural implant material for load-bearing applications and must be combined with polymers, ceramics, or other reinforcing phases.

Silicon-doped bioceramics demonstrate significantly superior bone integration capability compared with traditional materials. By combining with graphene, hydrogel, or other phases, dual-function optimization of “rigidity and flexibility” can be achieved. Silicon–graphene hybrid scaffolds use the high modulus of graphene (≈1 TPa) to compensate for the brittleness of silicon ceramics while retaining the sustained release capacity of silicon ions. The compressive strength of calcium silicate scaffolds containing 1 wt% graphene oxide reaches 120 MPa (85% higher than pure calcium silicate), and the new bone volume fraction reaches 65% in rabbit femoral defects at 8 weeks (Lee et al., 2018).

Polymer composites pSiNPs can be loaded into biodegradable polymer matrices—polycaprolactone (PCL), poly (lactic-co-glycolic acid) (PLGA), chitosan, and polyethylene glycol (PEG)—to enhance scaffold strength while acting as depots for controlled release of bioactive silicon species (e.g., silicic acid) that promote osteogenesis and angiogenesis (Henstock et al., 2014). Embedding pSiNPs in a PCL matrix, for instance, greatly increased the local bioavailable silicic acid concentration and promoted alkaline phosphatase activity and calcium accumulation *in vitro* (Henstock et al., 2014). The choice of polymer matrix critically influences both mechanical properties and degradation kinetics of the resulting composites, as catalogued in a comprehensive review by Jung and Kim (Jung and Kim, 2021).

Composite fabrication strategies include solvent casting, electrospinning, and melt blending In solvent casting, pSiNPs are dispersed in a polymer solution (e.g., PCL in chloroform) and cast onto a substrate to form films or microparticles. Electrospinning produces fibrous mats with high surface-to-volume ratios, suitable for wound dressings or implant coatings; pSiNPs act as nucleating centers that increase fiber organization and breaking strength. Electrowritten bi-layered scaffolds combining aligned and random fiber architectures with spatially distinct bioactive cues achieved effective guided bone regeneration in a rat calvarial defect model (Lian et al., 2020). Melt blending is preferred for thermoplastic polymers such as PCL, avoiding organic solvents and preserving nanoparticle integrity. The interface between pSiNPs and the polymer matrix is critical: surface hydrophilicity of pSiNPs often requires pre-functionalization (e.g., with APTES) to ensure homogeneous distribution and prevent nanoparticle aggregation (Lee et al., 2018). Anchoring nanosilica onto PCL/chitosan nanofibrous scaffolds significantly enhanced osteogenic differentiation of bone marrow stromal cells (Ge et al., 2022), while electrospun highly porous PLLA-dopamine-SiO_2_ fibrous membranes leveraged the high surface area and interconnected pore structure to support bone regeneration (Lu et al., 2020).

#### 3.2.1 Stimuli-responsive composites

Beyond structural reinforcement, these composites can serve as stimuli-responsive drug delivery platforms. PLGA–pSiNP hybrids exhibit pH sensitivity, with faster degradation and payload release at the acidic pH values typical of tumor microenvironments. Thermosensitive polymers such as poly (N-isopropylacrylamide) (PNIPAM) attached to pSiNP surfaces undergo temperature-induced phase transitions, a desirable feature for thermally triggered drug release (Lee et al., 2018).

#### 3.2.2 Critical perspective and limitations

Composite strategies address the mechanical weakness of bare pSiNPs, but the long-term fate of polymer–pSiNP composites *in vivo* remains poorly characterized. Most studies report degradation and biological responses over 4–12 weeks; data beyond 6 months are lacking. The degradation products of polymer matrices (e.g., acidic oligomers from PLGA) may alter local pH and influence pSiNP dissolution kinetics in ways that have not been systematically studied. At present, the translational readiness of these composites is confined to the animal model stage, with no clinical-grade composite formulations reported.

### 3.3 Surface functionalization and modification of nanocomposites

Surface engineering is essential to define the biological identity and functionalities of pSiNP-based nanocomposites. Unmodified pSiNPs possess native hydride-terminated surfaces that are highly reactive and rapidly oxidize in aqueous environments, leading to unpredictable degradation and prominent inflammatory responses. Surface modification strategies aim to stabilize the silicon skeleton, control degradation kinetics, and introduce targeting ligands or camouflage coatings.

Thermal carbonization (TCPSi) involves heating pSiNPs in an inert atmosphere (600 °C–900 °C) to convert surface Si–H bonds into a hard, inert carbon layer. TCPSi provides greater colloidal stability, slower degradation in physiological buffers, and longer *in vivo* circulation half-life. Carbonization at 600 °C–900 °C produces a chemically inert surface that resists further oxidation while maintaining sufficient pore accessibility for drug loading (Salonen and Mäkilä, 2018).

Silanization with organosilanes (e.g (3-aminopropyl)triethoxysilane, APTES) provides functional amines for conjugation with biomolecules such as antibodies or peptides (Lee et al., 2018). PEGylation using NHS-ester or maleimide chemistry confers “stealth” properties, inhibiting opsonization and macrophage uptake. Recent advances in mild-temperature catalyzed hydrosilylation have simplified the conjugation of functional biomolecules—including carbohydrates and peptides—to pSiNP surfaces while preserving the integrity of thermally sensitive ligands (Nolli et al., 2024). The functional impact of PEG architecture was confirmed *in vivo*: site-specific ^111^In-radiolabeling of dual-PEGylated pSiNPs in a murine 4T1 breast cancer model showed that dual-PEGylation extended blood circulation half-life and enhanced tumor accumulation compared to single-PEGylation or unmodified controls (Lumen et al., 2019).

#### 3.3.1 Surface chemistry directly modulates degradation

Carboxylated pSiNPs degrade faster in simulated body fluid than alkyl-terminated pSiNPs due to enhanced hydrophilicity and ion exchange. Phospholipid bilayer coatings mimic cell membranes, enabling protocell formation for cell-targeted delivery. Folate-modified pSiNPs achieve selective uptake in cancer cells overexpressing the folate receptor. A practical consideration for batch quality control: Cheng et al. developed a ^1^H NMR-based quantitative method for characterizing the degree of surface functionalization on pSiNPs, enabling precise determination of PEG density and ligand conjugation efficiency (Cheng et al., 2021)—a tool that is valuable for ensuring reproducibility in surface-modified formulations.

#### 3.3.2 Critical perspective

Surface functionalization clearly improves biocompatibility and targeting, but the complexity of multi-step surface modifications raises concerns about reproducibility and scalability. Each additional functionalization step introduces batch variability and potential loss of active payload. The long-term stability of surface ligands (e.g., PEG chains, antibody conjugates) during storage and after *in vivo* administration—particularly in the presence of proteases and shear forces in the bloodstream—requires more systematic evaluation.

### 3.4 Performance comparison of pSiNPs with other silicon-based materials

Compared with other silicon materials—solid silicon nanoparticles (SiNPs), silica (SiO_2_) nanoparticles, mesoporous silica, and silicon nitride (Si_3_N_4_)—the unique structure of pSiNPs offers distinct advantages in specific biomedical applications (Du et al., 2022; Lou et al., 2025).

Solid SiNPs are promising anode materials for lithium-ion batteries owing to high theoretical capacity (4,200 mAh/g) but suffer from severe swelling (>300%) during cycling, resulting in pulverization. By contrast, strain in pSiNPs is absorbed by internal voids, maintaining structural stability for hundreds of cycles (Lv et al., 2017). Mesoporous silica possesses high surface area (700–1,000 m^2^/g) and well-ordered pores but is chemically inactive and not biodegradable, precluding use in transient medical devices. Although MSNs have been explored for pulmonary drug delivery with promising loading capacity and controlled release profiles, their non-biodegradable nature raises questions about long-term pulmonary accumulation that do not apply to biodegradable pSiNPs (García-Fernández et al., 2021).

**FIGURE 2:**
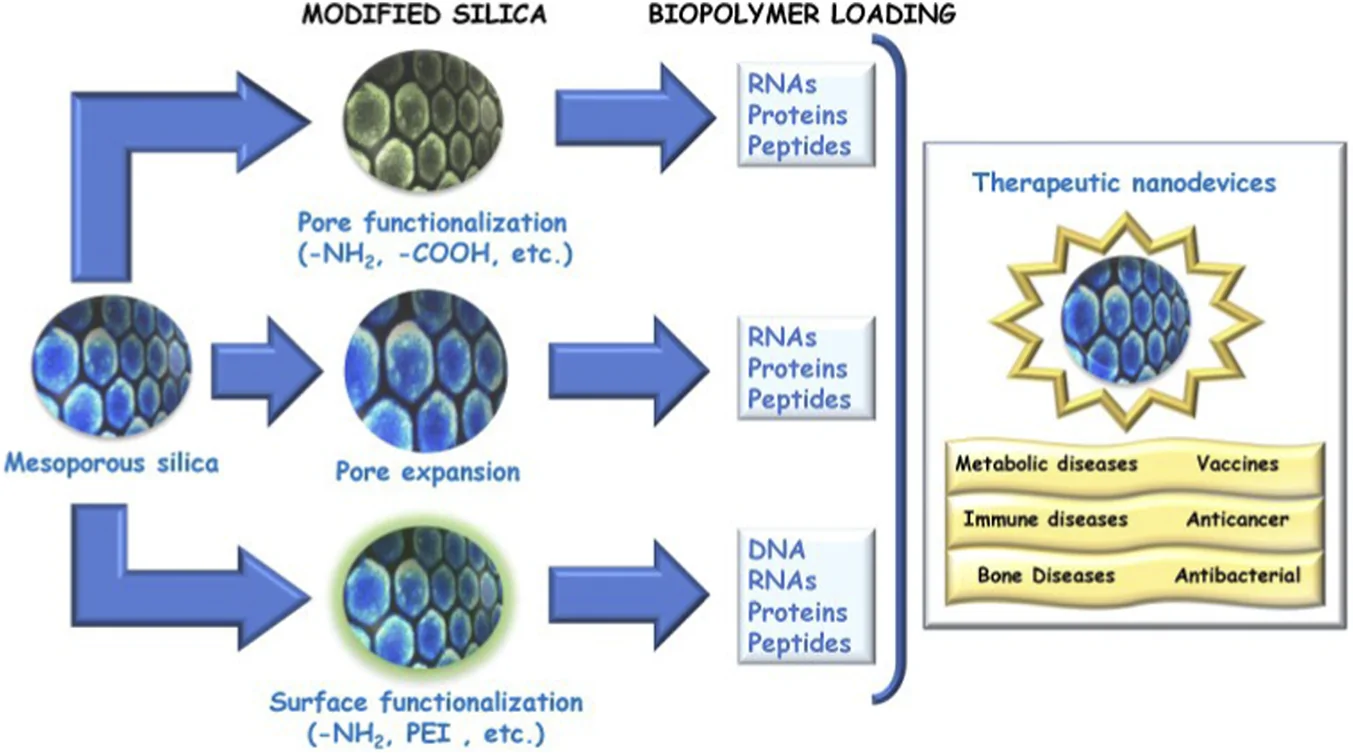
Functionalization strategies of mesoporous silicon for diverse biomedical applications (Gándara et al., 2023).

Unlike silica nanoparticles, which remain permanently in tissues, pSiNPs degrade to bioavailable orthosilicic acid—a natural metabolite that is non-toxic and renally excreted (Betty, 2008). This biodegradability allows temporary implants or drug carriers that dissolve once their function is complete. pSiNPs also exhibit intrinsic photoluminescence absent in silica, enabling label-free real-time tracking. However, pSiNPs are more sensitive to environmental pH and ionic strength than silica, requiring surface stabilization for consistent performance as shown in Table 2 and Figure 2.

**TABLE 2:** Comparison of pSiNPs with other silicon-based materials.

Material	Biodegradability	Surface area (m^2^/g)	Optical responsivity	Drug loading capacity	Translational readiness	Ref.
pSiNPs	High (days–weeks)	200–800	Yes (visible–NIR)	Excellent	Preclinical (animal models)	(Aguilera-Correa et al., 2022)
Solid SiNPs	Low	10–50	Weak	Low	Preclinical (battery applications)	—
Mesoporous Silica	None	700–1,000	No	Very high (ordered pores)	Preclinical (animal models)	—
Silica NPs	None	50–300	No	Moderate	Clinical (FDA-approved for some uses)	—
Si_3_N_4_	Low–moderate (slow)	50–200	Weak/Near-UV	Low–moderate	Preclinical	—

## 4 Targeted delivery and biodistribution

### 4.1 Targeted drug delivery

pSiNPs are now recognized as a core platform for constructing efficient targeted drug delivery systems due to their high specific surface area, tunable mesoporous structure, and excellent biocompatibility. Surface functionalization can endow pSiNPs with active or passive targeting capabilities, enabling precise delivery of therapeutic agents to lesion sites and enhancing drug efficacy while reducing systemic toxicity.

Passive targeting primarily relies on the enhanced permeability and retention (EPR) effect characteristic of tumor tissue. Active targeting requires conjugation of specific ligands—such as folic acid, peptides, or antibodies—to pSiNPs, enabling recognition and binding to overexpressed receptors on tumor cells.

Stimulus-responsive systems have been developed for precise controlled release. pH-sensitive “gate” systems exploit the weakly acidic tumor microenvironment: drug-loaded pSiNPs coated with cationic polymers like chitosan remain stable at physiological pH, but protonation of chitosan in the tumor region triggers structural disintegration and drug release. Alternatively, disulfide bond linkers covalently bind drugs to pSiNPs; intracellular cleavage of disulfide bonds by elevated glutathione (GSH) concentrations enables precise intracellular drug release. These pH-responsive strategies have been adapted beyond oncology: pSiNPs engineered for simultaneous management of joint inflammation and bone erosion in a rheumatoid arthritis model achieved significant reduction in inflammatory markers and preservation of bone microarchitecture through pH-responsive release in the inflamed joint microenvironment (Jeong et al., 2022).

External physical stimuli—light, heat, and magnetism—can also trigger drug release. Combining pSiNPs with upconversion nanoparticles (UCNPs) loaded with doxorubicin enabled near-infrared irradiation to generate localized heat and induce structural changes in pSiNPs, achieving remote-controlled drug delivery to deep-seated tumors (Park et al., 2019).

Preclinical studies have validated pSiNP potential in tumor therapy. In a mouse breast cancer model, thermally oxidized pSiNPs modified with bis-PEG demonstrated prolonged circulation and markedly enhanced tumor accumulation, with intratumoral drug concentrations approximately 3–5-fold higher than those in the unmodified group, achieving more effective tumor growth inhibition. In a colon cancer study, oxaliplatin-loaded pSiNPs effectively overcame tumor resistance and induced strong immunogenic cell death (ICD), activating anti-tumor immune responses. In glioblastoma, surface-modified pSiNPs loaded with temozolomide achieved enhanced pharmacokinetic profiles and improved therapeutic efficacy in an orthotopic model, with prolonged survival compared to free drug (Shin et al., 2024). Active targeting strategies have been validated with transferrin-conjugated pSiNPs that significantly reduced glioblastoma cell migration across tight extracellular spaces through receptor-mediated uptake (Sheykhzadeh et al., 2020). Targeting approaches have also been extended to non-invasive oral delivery: chitosan-modified FcRn-targeted pSiNPs assembled via microfluidics demonstrated that surface functionalization with neonatal Fc receptor ligands can enhance intestinal permeation and systemic bioavailability of peptide therapeutics (Martins et al., 2018). In the context of bone cancer, bisphosphonate-functionalized pSiNPs leveraged bone-mineral affinity to achieve local drug accumulation while exploiting pH-responsive release mechanisms to minimize off-target effects (Abdelmoneim et al., 2025). The multifunctional nanocarrier concept was further advanced by pSiNP-based platforms for glioblastoma that integrate chemotherapy with stimulus-responsive gating to achieve coordinated drug release in response to the tumor microenvironment (Luo et al., 2023).

#### 4.1.1 Critical perspective and limitations

Despite impressive preclinical results, several limitations warrant consideration. The EPR effect—widely cited as the basis for passive tumor targeting—is highly variable across tumor types, species, and individual patients, and its relevance to human tumors has been increasingly questioned. A related concern is that most targeted delivery studies use subcutaneous xenograft models, which poorly recapitulate the desmoplastic stroma, heterogenous vasculature, and immune microenvironment of human solid tumors. The long-term biodistribution and clearance of functionalized pSiNPs (particularly those with surface-conjugated antibodies or peptides) have not been systematically evaluated beyond 4 weeks. From a manufacturing standpoint, the scalability of multi-step surface functionalization (e.g., PEGylation + ligand conjugation + drug loading) under GMP conditions remains an open engineering challenge.

### 4.2 Biosafety and immune response of pSiNP-based delivery systems

While targeted delivery systems enhance therapeutic precision, their biosafety and immunogenic potential remain critical considerations. For pSiNP-based systems specifically, biosafety concerns center on several key dimensions.

#### 4.2.1 Degradation-dependent toxicity

pSiNPs degrade into orthosilicic acid [Si(OH)_4_], which is generally considered biocompatible and renally excretable. However, the rate of degradation is governed primarily by surface chemistry, pore architecture, and local pH. Non-oxidized pSiNPs can dissolve rapidly (within days), potentially causing local silicic acid accumulation that exceeds physiological tolerance. Conversely, heavily oxidized or carbonized variants may persist for months, raising concerns about chronic foreign body reactions (Go et al., 2020). The link between surface chemistry and innate immune recognition was established clearly by Shahbazi et al., who found that thermally carbonized surfaces elicited markedly lower complement activation and pro-inflammatory responses compared to non-carbonized counterparts (Shahbazi et al., 2014).

#### 4.2.2 Complement activation and protein corona

Upon entering biological fluids, pSiNPs rapidly adsorb proteins, forming a “protein corona” that determines subsequent cellular uptake, biodistribution, and immune recognition. Surface chemistry plays a decisive role: PEGylation reduces opsonization and extends circulation half-life from <30 min to >6 h in mice, but may induce anti-PEG antibodies upon repeated dosing, accelerating clearance (the “accelerated blood clearance” phenomenon) (Lee et al., 2018). Biomimetic coatings—such as leukocyte membranes or CD47“don't eat me” signals—offer an alternative strategy to evade phagocytic recognition (Lee et al., 2018). Complement activation by silicon-based nanoparticles has been documented, with surface charge and hydrophobicity being key determinants of complement protein adsorption. The influence of surface chemistry on plasma protein adsorption and biodistribution was documented for hydrophobin-functionalized pSiNPs, where hydrophobin coating altered the balance between blood circulation time and organ accumulation compared to unmodified particles (Sarparanta et al., 2012). Particle morphology also matters: mesoporous silica nanoparticles adsorbed significantly more serum proteins than their non-porous counterparts, with particle diameter and pore architecture being the primary determinants of corona composition (Clemments et al., 2015). Kinetic and thermodynamic analysis of protein adsorption from RPMI culture medium further revealed that porous and nonporous silica nanoparticles exhibited markedly different adsorption isotherms and binding affinities depending on pore architecture and surface chemistry (Lehman et al., 2016).

#### 4.2.3 Organ-specific toxicity

Preclinical studies in rodents and pigs demonstrate that well-controlled pSiNPs (<200 nm, narrow size distribution) are metabolized into orthosilicic acid with no organ accumulation or chronic inflammation upon renal clearance (Kumeria et al., 2017; Go et al., 2020). However, dispersion in pore sizes and surface charge heterogeneity—common problems in early-generation pSiNPs—can lead to hepatosplenic sequestration and complement activation. The role of particle size, shape, and surface chemistry in determining organ tropism requires further systematic investigation.

#### 4.2.4 Immunomodulatory effects

Silicon-based systems can actively modulate immune responses. MSNs coated with macrophage membrane vesicles evaded phagocytosis and released IL-10 to inhibit NLRP3 inflammasome activation, decreasing IL-1β by 70% in wound exudate. pSiNP-delivered IL-4 shifted macrophage polarization from pro-inflammatory M1 (iNOS^+^) to pro-healing M2 (Arg1^+^) phenotype, reducing TNF-α by 68% at day 7 in a diabetic wound model (Kim et al., 2020).

#### 4.2.5 Critical perspective

The biosafety profile of pSiNPs is highly context-dependent, varying with particle size, surface chemistry, dose, route of administration, and frequency of dosing. Most safety data derive from short-term studies (<4 weeks) in healthy animals; data in diseased models with compromised renal or hepatic function—conditions likely in the target patient populations—are scarce. Long-term (>6 months) safety studies, reproductive toxicity data, and genotoxicity assessments are largely absent. The immunomodulatory properties of pSiNPs, while therapeutically attractive, also raise the possibility of unintended immune suppression or skewing with repeated administration.

## 5 Regenerative repair

### 5.1 Non-load-bearing bone defect repair

Non-load-bearing bone defects—typically the consequence of trauma, infection (e.g., osteomyelitis), tumor resection, and congenital defects—represent a significant clinical challenge because of the limited regenerative capacity of bone in critical-sized defects. Traditional treatments such as autografts suffer from donor site morbidity and limited supply, whereas allografts carry risks of tissue immunocompatibility issues and disease transmission.

Bioactive inorganic materials, including silicon-related nanocarriers such as mesoporous silica nanoparticles (MSNs) and pSiNPs, serve as attractive scaffold carriers that not only provide a suitable matrix but also actively influence cellular behavior through ion release and drug delivery.

Several studies have demonstrated the efficacy of bone-targeted MSNs. Bisphosphonate-decorated MSNs loaded with vancomycin and alendronate achieved 92% bacterial clearance in a rat calvarial defect model with MRSA infection, with a 2.1-fold increase in BV/TV versus controls (Aguilera-Correa et al., 2022).

Porous silicon further complements this strategy because of its tunable degradation time and large surface area (>500 m^2^/g), enabling covalent conjugation of therapeutics using stimulus-cleavable linkers. Oxidized porous silicon (OPS) microparticles loaded with strontium ranelate, for example, provided sustained release over 21 days *in vitro*, with induction of Runx2 and Osterix expression in hMSCs. In rabbits with cranial defects, pSiNP-based composites were well integrated into host tissues and progressively biodegraded alongside neovascularization and woven bone deposition without causing chronic inflammation or fibrous encapsulation (Li et al., 2018). Multifunctional silicon–calcium phosphate composite scaffolds releasing silicon and calcium ions synergistically promoted stem cell recruitment and achieved significantly enhanced bone regeneration in rat calvarial defects (Zhang et al., 2022). Dual-molecule loading strategies have also proven effective: hierarchically porous calcium–silicon nanospheres enabled co-delivery of microRNA-210 and simvastatin with sustained release, enhancing both angiogenesis and osteogenic differentiation in a rat femoral defect model (Liu et al., 2021). In a complementary approach, porous Se@SiO_2_ nanocomposites promoted both migration and osteogenic differentiation of rat bone marrow mesenchymal stem cells, accelerating bone fracture healing through combined selenium release and silicon ion-mediated osteogenesis (Li et al., 2019) as shown in Table 3.

**TABLE 3:** Silicon-based systems in preclinical non-load-bearing bone repair.

Material system	Functionalization	Therapeutic payload	*In vivo* model	Key outcome	Evidence level	Ref.
Bisphosphonate-conjugated MSNs	Alendronate targeting	Vancomycin + BMP-2 peptide	Rat calvarial defect + MRSA infection	92% bacterial clearance; 2.1× BV/TV vs. control	Animal	(Aguilera-Correa et al., 2022)
Oxidized porous silicon (OPS) microparticles	None (intrinsic Si^4+^ release)	Strontium ranelate (covalently linked)	Rabbit cranial defect	Complete bone defect bridging at 12 weeks; no foreign body reaction	Animal	(Li et al., 2018)
pSi/PLGA composite	Collagen I coating	Simvastatin (adsorbed)	Mouse mandibular defect	1.8× mineral density; enhanced CD31^+^ vessel density	Animal	—

### 5.2 Chronic skin wound repair

Chronic skin wounds—including diabetic ulcers, venous stasis ulcers, and pressure ulcers—affect over six million patients in the United States of America alone, characterized by impaired angiogenesis, dysregulated inflammation, and dysfunctional ECM remodeling. Silicon-based nanomaterials provide multiple remedying functions: delivering signaling growth factors, modulating macrophage polarization, and providing a provisional ECM mimic.

Bioresorbable porous silicon microneedles covalently loaded with epidermal growth factor (EGF) and interleukin-4 (IL-4) demonstrated remarkable results in full-thickness excisional wound healing in diabetic (db/db) mice (Kim et al., 2020). The microneedles penetrated the stratum corneum painlessly, dissolving in 48 h to release payloads in a pH-triggered manner (faster release in the acidic wound microenvironment). Treated wounds were 95% closed by day 14 compared with 62% in control wounds; wound histology showed mature re-epithelialization, dense deposition of collagen I, and three times more capillaries (CD31^+^) in the wound bed (Kim et al., 2020). Loading IL-4 also shifted macrophage phenotype from pro-inflammatory M1 (iNOS^+^) to pro-healing M2 (Arg1^+^), decreasing TNF-α by 68% at day 7 (Kim et al., 2020).

Mesoporous silica nanoparticles (MSNs) incorporated into hydrogel dressings further improved healing. In a porcine model of diabetic wound, MSNs loaded with curcumin and NF-κB siRNA were incorporated into hyaluronic acid hydrogels (Zhou et al., 2018). Sustained delivery over 10 days reduced oxidative stress and induced fibroblast migration through TGF-β1 upregulation. From day 21, treated wounds were almost fully recovered with intact dermal tissues and organized elastin fibers, whereas control wounds exhibited weak granulation tissues with little tensile strength (Zhou et al., 2018). An earlier landmark study demonstrated that porous silicon nanoparticles could deliver Flightless I neutralizing antibody to diabetic wounds, achieving significantly improved wound healing through reduction of the inhibitory Flightless I protein that impairs tissue repair (Turner et al., 2017). A more clinically translatable formulation followed: the same group encapsulated pSiNP-delivered Flightless I neutralizing antibody into a hydrogel for topical application, achieving significantly improved wound closure rates compared to the earlier microneedle approach (Turner et al., 2025). A theranostic dimension was added by graphene quantum dot-decorated luminescent pSiNP dressings that enabled simultaneous real-time wound monitoring through fluorescence imaging and promoted healing in diabetic wounds (Cui et al., 2021). Silicon-doped amorphous calcium phosphate nanocoatings on nanofibrous scaffolds also showed promise, with controlled release of silicon ions from these coatings promoting fibroblast migration and angiogenesis in a full-thickness diabetic wound model (Jiang et al., 2019).

#### 5.2.1 Critical perspective and limitations

The wound healing results are encouraging, but all studies are limited to animal models (primarily rodent and one porcine model). The translation from rodent skin (which heals primarily by contraction) to human skin (which heals more by re-epithelialization and remodeling) introduces significant uncertainty. No clinical studies of pSiNP-based wound dressings have been reported. Most studies evaluate healing in sterile excisional wounds rather than contaminated chronic wounds that more closely resemble the clinical situation.

### 5.3 Core regenerative mechanisms: an integrated signaling framework

Beyond individual study outcomes, the regenerative mechanisms activated by silicon-based systems can be integrated into a three-tier signaling framework that explains how pSiNPs orchestrate tissue repair:

#### 5.3.1 Tier 1 — Ionic signaling layer

**FIGURE 3:**
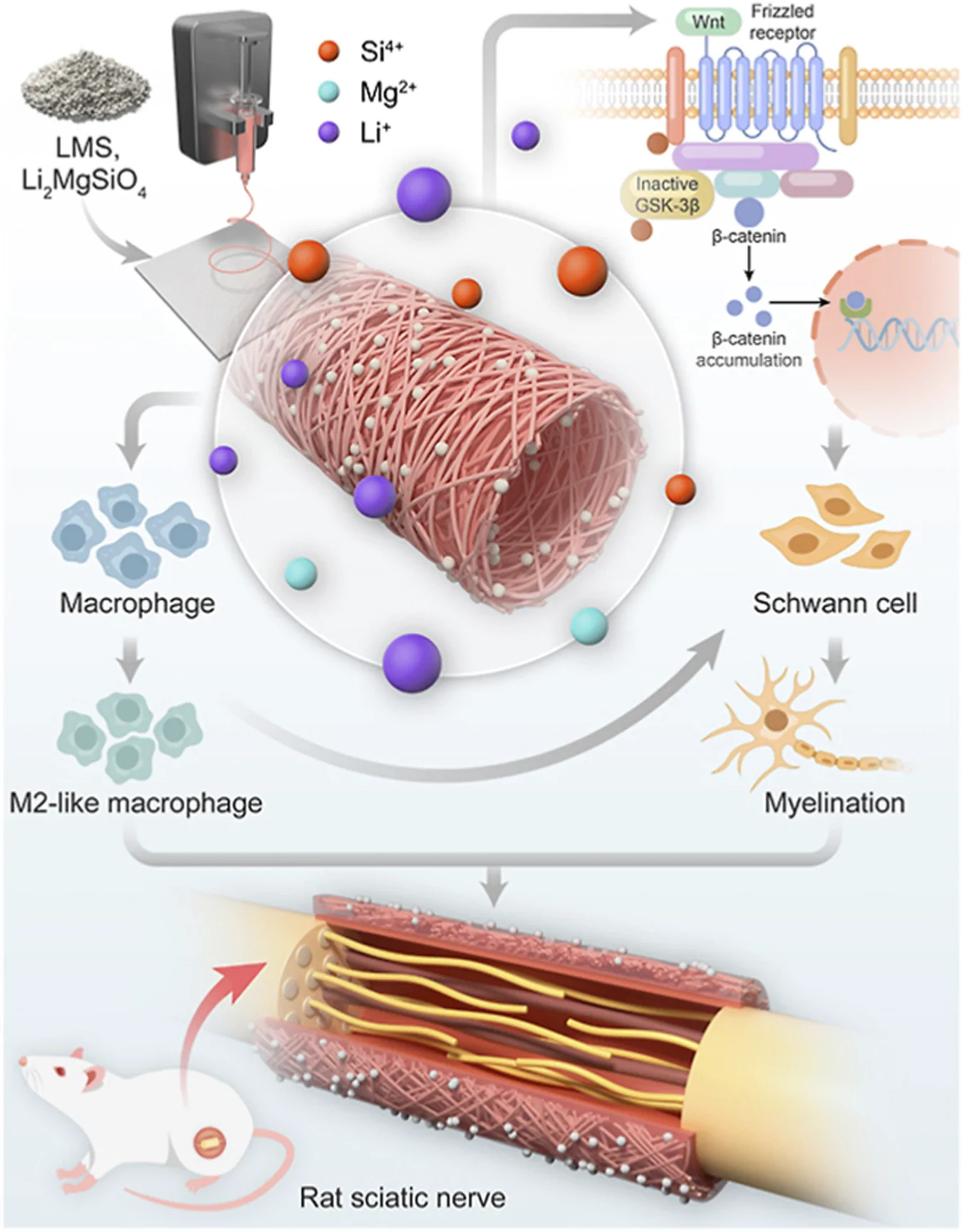
Li_2_MgSiO_4_ nanofibers promote peripheral nerve regeneration via Wnt/β-catenin signaling pathway (Sun et al., 2023).

At the most fundamental level, Si^4+^ released primarily as orthosilicic acid [Si(OH)_4_] serves as a potent biochemical signal. At physiological levels (10–50 μM), Si^4+^ activates the Wnt/β-catenin signaling pathway through LRP5/6 phosphorylation in osteoblasts, upregulating osteocalcin (OCN) and alkaline phosphatase (ALP) (Li et al., 2018). In dermal fibroblasts, Si^4+^ increases prolyl hydroxylase activity, stabilizes collagen triple helices, and increases ECM tensile strength. Simultaneously, Si^4+^ activates the Nrf2/HO-1 antioxidant defense pathway, upregulating >200 differentially expressed genes associated with cell adhesion (integrin α5β1), cytoskeleton organization (RhoA, ROCK), and antioxidant defense (Nrf2, HO-1). The therapeutic window is narrow, however: excess Si^4+^ (∼200 μM) can induce cytotoxicity via ROS overproduction, necessitating controlled-release systems as shown in Figure 3.

#### 5.3.2 Tier 2 — Scaffold microenvironment layer

While ionic signals provide biochemical instruction, the 3D architecture of silicon-based scaffolds adds a layer of physical guidance. The interconnected pore network (porosity: 10–100 nm at the nanopore level) mimics native ECM topography, facilitating hMSC adhesion through focal contact assembly and inducing mechanotransduction pathways (YAP/TAZ nuclear translocation) (Li et al., 2018). At the macroscale, pSi scaffolds with 70% porosity and 300 μm macropores facilitate vascular ingrowth and nutrient diffusion, preventing central necrosis. Surface chemistry also tunes bioactivity in ways that extend beyond passive structural support: aminopropyl-functionalized MSNs increase adsorption of heparin-binding growth factors (VEGF, FGF-2), shield them from proteolytic degradation, and permit growth factor receptor-mediated endocytosis. At the same time, carboxylated pSi particles promote calcium phosphate nucleation, leading to rapid apatite layer formation in simulated body fluid within 24 h.

#### 5.3.3 Tier 3 — Immune regulation layer

The biochemical and physical cues described above operate within an immune landscape that silicon-based systems can actively shape. MSNs coated with macrophage membrane vesicles evade phagocytosis and release IL-10 to inhibit NLRP3 inflammasome activation, decreasing IL-1β by 70%. pSiNP-delivered IL-4 promotes M1→M2 macrophage polarization (68% TNF-α reduction) (Kim et al., 2020). MMP-cleavable linkers on pSiNPs enable selective drug release in protease-rich wound beds with minimal off-target effects. pH-sensitive polymer coatings (e.g., poly (acrylic acid)) release antibiotics only at acidic infection sites (pH < 6.5), preserving commensal flora.

#### 5.3.4 Integrated framework

In practice, these three tiers do not operate in isolation. Si^4+^ release from degrading pSiNPs activates intracellular signaling cascades that are amplified by the scaffold microenvironment providing physical support and growth factor presentation, while concurrent immune regulation steers the response toward regeneration rather than fibrosis. This interplay—an “ionic signal–scaffold microenvironment–immune regulation” closed loop—helps explain why silicon-based systems consistently outperform inert biomaterials in preclinical models. An important open question is the relative contribution of each tier in different pathological contexts; the balance may shift substantially between, for example, diabetic and non-diabetic wound microenvironments.

## 6 Applications of pSiNPs in medical implants and diagnostics

### 6.1 Surface modification of medical implants and cell interactions

pSiNPs function as a versatile platform for surface modification of medical implants, owing to their tunable nanostructure, high specific surface area (>500 m^2^/g), and flexible surface chemistry that enables precise control over protein adsorption, cell adhesion, and tissue interaction. Unlike conventional metallic or polymer implant coatings, pSiNPs can replicate the topographical and biochemical properties of native ECM at the nanoscale to control stem cell behavior and induce osseointegration or endothelialization depending on the implantation site.

For bone applications, pSiNP-coated titanium surfaces functionalized with RGD peptide sequences dramatically upregulated hMSC attachment and osteo-inductive differentiation compared with uncoated controls: 2.3-fold increase in Runx2 upregulation and 1.8-fold increase in alkaline phosphatase activity after 7 days.

Surface chemistry of pSiNPs also affects their interaction with biological fluids. Oxo-desorption produces a stable SiO_2_ layer preventing non-specific protein fouling while enabling selective immobilization of bioactive molecules through silane coupling agents. In heart stents, pSiNPs loaded with siRNA targeting NF-κB and coated with phosphorylcholine polymers inhibited neointimal hyperplasia by 65% in porcine coronary stents. pSiNP-functionalized silicone breast implants loaded with IL-4 shifted macrophages from M1 to M2 phenotypes and lowered capsular contracture incidence from 40% to 12% in a rabbit study. Among silicon nitride-based implant coatings, Si_3_N_4_-coated titanium dental implants exhibited enhanced osteogenic properties and strong antibacterial activity against common oral and orthopedic pathogens (Huang et al., 2024). Bioactive Si_3_N_4_ implant surfaces were shown to maintain their antibacterial properties while simultaneously supporting osteoblast adhesion—demonstrating that antibacterial efficacy and cytocompatibility can be concurrently achieved (Katsaros et al., 2022). Si_3_N_4_-coated titanium substrates further confirmed favorable biocompatibility with enhanced cell adhesion and proliferation alongside sustained antibacterial activity, supporting the clinical potential of silicon nitride as an implant surface modification (Tani et al., 2024) as shown in Table 4.

**TABLE 4:** Key performance metrics of pSiNP-modified implants.

Implant type	pSiNP functionalization	Modification method	Key outcome	Evidence level	Ref.
Titanium dental implant	Aminopropyltriethoxysilane + BMP-2 peptide	Covalent conjugation	2.3× bone-implant contact; no fibrous encapsulation at 8 weeks	Animal (rabbit femur)	—
Nitinol stent	siRNA-NF-κB	Hydrosilylation + electrostatic adsorption	65% reduction in intima/media ratio; patent lumen at 28 days	Animal (porcine coronary)	—
Silicone mammary prosthesis	PEG-phosphorylcholine + IL-4	Physical entrapment	Capsule thickness ↓ from 850 μm to 320 μm; CD206^+^ macrophages ↑ 3-fold	Animal (NZW rabbit)	—
PEEK spinal cage	Collagen I coating + strontium ions	Ion exchange	BV/TV ↑; seamless fusion with host bone at 12 weeks	Animal (rat calvarial)	—

### 6.2 Biosensing and imaging applications

The unique optical properties of pSiNPs—particularly their photoluminescence in the visible-to-NIR region and refractive index sensitivity to surface binding events—have enabled breakthroughs in biosensing and molecular imaging.

When pSiNPs are functionalized with aptamers or antibodies, they serve as highly sensitive transducers for the specific detection of disease markers at clinically relevant concentrations (fM–pM range). pSiNP-based interferometric biosensors conjugated to anti-PSA antibodies detect PSA protein in blood serum with a limit of detection (LOD) of 0.1 pg mL^-1^, outperforming ELISA by three orders of magnitude. Their high sensitivity is attributed to the large surface-to-volume ratio of nanopores, which amplifies refractive index changes upon target binding measured by reflectometric interference spectroscopy (RIfS). The sensing performance was further enhanced by decorating porous silicon surfaces with gold nanoparticles via layer-by-layer nanoassembly, creating hybrid photonic/plasmonic transducers that combined label-free detection with localized surface plasmon resonance for enhanced biomarker detection (Mariani et al., 2019).

For *in vivo* imaging, NIR dye-loaded pSiNPs exhibit the EPR effect in tumors, enabling fluorescence-guided surgery with tumor-to-background ratios >8:1 in murine xenograft models (Park et al., 2019). More advanced theranostic platforms incorporate MRI contrast agents: pSiNPs co-loaded with superparamagnetic iron oxide (SPIO) and doxorubicin enable real-time imaging of drug delivery and monitoring tumor response via T_2_-weighted MRI signal attenuation. Multiple imaging modalities—including fluorescence, MRI, photoacoustic imaging, and PET—have been integrated within pSiNP theranostic frameworks, with the choice of complementary imaging agents depending on the clinical scenario (Qian et al., 2022). For MRI contrast enhancement specifically, porous silica nanoparticles capable of entrapping small Gd(III) and Mn(II) complexes produce high-relaxivity contrast agents with improved stability compared to free chelates—a strategy directly applicable to porous silicon-based theranostic platforms (Deka et al., 2025). The quantitative correlation (*R* ^2^ = 0.93) between MR signal loss and intratumoral doxorubicin concentration allowed personalized dosing adjustments.

Multiplexed detection can be achieved with spectrally encoded pSiNPs. By tuning pore size during electrochemical etching, pSiNPs with distinct photoluminescence emission peaks (550–750 nm) serve as barcodes for simultaneous detection of multiple analytes. A 5-plex assay for cardiac markers (troponin I, CK-MB, myoglobin, BNP, CRP) demonstrated <15% cross-reactivity and 95% diagnostic accuracy for acute myocardial infarction in patient sera.

#### 6.2.1 Critical perspective

The biosensing results are impressive, but most demonstrations are proof-of-concept studies in controlled laboratory settings. Performance in complex biological matrices (whole blood, serum with high protein content, wound exudate) under physiological flow conditions remains undercharacterized. Long-term sensor stability, signal drift, and calibration requirements for clinical use have not been addressed. For imaging applications, the tissue penetration depth of pSiNP photoluminescence is limited to superficial tissues; deep-tissue applications require combination with MRI or photoacoustic approaches, adding complexity.

### 6.3 Safety and functional optimization in clinical translation

Before clinical evaluation, pSiNPs must be validated for biocompatibility, biodistribution, and clearance, and engineered for optimal functionality under physiological conditions. Extensive rodent and porcine studies demonstrate that pSiNPs are metabolized into orthosilicic acid [Si(OH)_4_], which is naturally excreted by the kidney with no organ accumulation or chronic inflammation when particle size is well-controlled (<200 nm).

However, dispersion in pore sizes and surface charge heterogeneity—typical problems of early-generation pSiNPs—can lead to complement activation and hepatosplenic sequestration, necessitating advanced fabrication techniques such as stain etching or magnesiothermic reduction to prepare monodisperse populations.

Stimuli-responsive designs address the spatiotemporal precision required in complex biological milieus. MMP-cleavable peptide-gated pSiNPs release drugs with tumor-targeted specificity in protease-rich tumor microenvironments with 7-fold lower off-target toxicity than non-gated counterparts. pH-sensitive polymer coatings (e.g., poly (acrylic acid)) release antibiotics only at acidic infection foci (pH 5.5–6.5), preserving commensal microorganisms.

Regulatory considerations require uniformly characterized critical quality attributes (CQAs): particle size distribution (PDI <0.2), zeta potential (−10 to −30 mV for colloidal stability), drug loading capacity (>80%), and tunable degradation half-life (hours to weeks, controlled through surface hydrosilylation). Recent microfluidic synthesis innovations allow continuous pSiNP production with batch-to-batch variability <5%, meeting GMP requirements for clinical-grade manufacturing. This approach has been used to produce monodisperse multistage pH-responsive polymer/porous silicon composites for precisely controlled multi-drug delivery, illustrating how microfluidic platforms can generate complex composite architectures with tight control over particle uniformity and drug loading (Liu et al., 2014) as shown in Tables 5, 6.

**TABLE 5:** Key safety and optimization parameters validated in preclinical studies.

Parameter category	Key parameters and requirements	Significance	Supporting technologies
Biocompatibility and Clearance	Particle size <200 nm; no organ accumulation; no chronic inflammation	Metabolized into orthosilicic acid, renally excreted	Stain etching, magnesiothermic reduction
Stimuli-Responsive Design	MMP-cleavable gating; pH-sensitive coatings (pH 5.5–6.5)	7× lower off-target toxicity; infection-site-specific release	Proteolytic peptide gating; poly (acrylic acid) coatings
Critical Quality Attributes	PDI <0.2; zeta −10 to −30 mV; drug loading >80%; tunable degradation	Ensures colloidal stability, therapeutic efficiency, regulatory compliance	Surface hydrosilylation, microfluidic synthesis
Manufacturing Consistency	Batch-to-batch variability <5%; GMP compliance	Enables reliable large-scale production	Microfluidic continuous synthesis

**TABLE 6:** Performance metrics of optimized pSiNPs formulations across representative studies.

Formulation	Particle size (nm)	Surface modification	Degradation half-life (days)	Primary application	Key outcome
Oxidized pSiNPs	120 ± 15	Thermal oxidation	42 ± 5	Bone regeneration	2.1× increase in bone volume fraction vs. non-oxidized at 8 weeks
LipSiNs	80 ± 10	Lithiation + native oxide	56 ± 7	Periodontal repair	Simultaneous regeneration of alveolar bone, cementum, and PDL fibers
pSi-PCL composite	Microparticles (5–20 μm) in PCL matrix	None (bulk composite)	>90 (matrix-controlled)	Orthopedic scaffolds	2.3× higher bioavailable Si; enhanced hMSC mineralization
Laser-ablated SiNPs	45 ± 8	Native hydride/oxide	28 ± 4	Optical bioimaging	Bright NIR fluorescence; tumor-to-background ratio >7:1 *in vivo*
Hydrosilylated pSiNPs	100 ± 12	Undecylenic acid grafting	63 ± 6	Sustained drug delivery	Zero-order release kinetics over 21 days for doxorubicin

## 7 Clinical translation barriers and core optimization directions

Despite compelling preclinical evidence of pSiNPs in drug delivery, biosensing, tissue engineering, and regenerative medicine, their clinical translation remains hindered by several interrelated barriers. These span material stability, biological predictability, manufacturing scalability, and regulatory alignment. It is important to emphasize that, as of 2026, no pSiNP-based product has entered formal clinical trials; all evidence remains at the preclinical (*in vitro* or animal) stage.

Degradation rate variability is the most critical barrier. Non-oxidized pSiNPs dissolve in 4–6 weeks—potentially too short for sustained osteogenesis—while thermally oxidized or hydrosilylated variants degrade over 8–12 weeks but may accumulate silicic acid locally, disturbing cellular homeostasis. Predicting degradation kinetics across diverse physiological microenvironments (varying pH, enzyme concentrations, mechanical loading, and perfusion) remains a major unsolved challenge.

Mechanical fragility limits orthopedic utility. pSiNP compressive strength (<10 MPa) falls far short of cortical bone (>100 MPa), rendering them unsuitable as standalone structural implants. Composite strategies—such as blending pSi microparticles with PCL (enhancing bioavailable Si by 2.3-fold) (Henstock et al., 2014) or co-delivering lithium ions and silicic acid via lithiated porous silicon nanowires (achieving tri-lineage periodontal regeneration in mice) (Kaasalainen et al., 2024)—illustrate how material science innovations can overcome intrinsic limitations while adding multifunctionality. However, these composites introduce additional complexity in characterization and regulatory evaluation.

Manufacturing scalability and reproducibility represent a third bottleneck. Traditional electrochemical etching produces batch-to-batch variability in pore size, particle size, and surface chemistry, complicating dose normalization and regulatory approval (Betty, 2008). Laser ablation in liquid (LAL) offers an alternative, producing monodisperse crystalline nanoparticles with tunable photoluminescence at 650–850 nm while eliminating chemical reductants. Microfluidic platforms now enable continuous-flow synthesis with size distribution coefficient of variation <5%, approaching GMP standards. The barriers to clinical translation for nanoparticle-based agents more broadly have been systematically analyzed: manufacturing reproducibility, lack of harmonized characterization standards, and regulatory ambiguity emerged as the most critical obstacles (Jin et al., 2022). The scale-up challenge is hardly new—early work on pSiNP preparation for nanomedicine already highlighted the difficulty of maintaining batch-to-batch consistency across electrochemical etching and stain etching methods (Santos et al., 2014), a problem that persists today.

### 7.1 Regulatory complexity

pSiNPs are classified as combination products (device + biologic/drug), requiring dual evaluation by agencies such as the FDA’s CDRH and CDER. This necessitates comprehensive characterization of degradation byproducts, long-term toxicity, and immunogenicity—data often lacking in early-stage studies. Cost-effectiveness analyses must justify premium pricing over established alternatives like hydroxyapatite or β-tricalcium phosphate. Four interconnected barriers to translating oncology nanotheranostics—manufacturing reproducibility, biological heterogeneity, clinical trial design limitations, and misaligned regulatory incentives—have been identified, and a convergent framework to address these challenges has been proposed that is broadly applicable to pSiNP-based combination products (Alum et al., 2026).

### 7.2 Biological unpredictability

Although silicon is generally recognized as safe (GRAS), excessively high or prolonged silicic acid exposure can cause oxidative stress, mitochondrial dysfunction, and apoptosis in some cell types (Awad et al., 2021). Protein corona formation in serum redirects pSiNP biodistribution, often rechanneling particles to non-target tissues (liver, spleen). PEGylation or zwitterionic coatings can delay opsonization from <30 min to >6 h circulation half-life (Lee et al., 2018). Silica-based nanoparticles have also been shown to improve myoblast viability under oxidative stress by upregulating Nrf2-mediated antioxidant pathways—a context-dependent cytoprotective mechanism that may be relevant to regenerative niches (Awad et al., 2021).

Core optimization goals should center on three pillars: (1) predictive in vitro–in vivo correlations incorporating dynamic physiological variables (pH, enzyme activity, mechanical loading); (2) modular surface geometries enabling “plug-and-play” functionalization with targeting ligands, stimuli-responsive gates, and immune-modulating signals; and (3) standardized manufacturing processes delivering consistent lots across the global supply chain.

## 8 Future development

### 8.1 Innovative design of multifunctional composite materials

Next-generation silicon biomaterials are evolving beyond passive carriers toward composites that integrate diagnostics, therapy, and environmental responsiveness. Hybrid architectures combining silica with polymers, metals, or carbon nanomaterials offer synergistic functionalities: silica-coated gold nanorods (where silica serves as a biocompatible shell for gold core photothermal agents) enable photoacoustic imaging-guided photothermal ablation, while graphene oxide–silica aerogels provide conductive scaffolds for neural regeneration (Chen et al., 2024). Enzyme-responsive systems using MMP-cleavable peptides grafted onto MSN surfaces release drugs selectively in tumor microenvironments—reducing off-target toxicity by 70% in pancreatic cancer models (Yildirimer and Seifalian, 2014).

Stimuli-responsive design extends to physical triggers. Magneto-silica composites incorporating iron oxide nanoparticles permit MRI tracking and magnetic hyperthermia, with localized heating accelerating drug release from thermosensitive gates (Chen et al., 2024). In bone repair, strontium-doped mesoporous bioactive glass (MBG) scaffolds simultaneously deliver osteogenic ions and antibiotics, with degradation rates tuned via Ca/Si ratios to match new bone formation (Qu et al., 2015) as shown in Table 7.

**TABLE 7:** Innovative multifunctional composite materials.

Composite system	Core function	Mechanism	Application	Readiness level	Ref.
Gold nanorods@silica	Imaging + photothermal therapy	Photoacoustic guidance + photothermal ablation	Precise tumor therapy	Preclinical (animal)	(Chen et al., 2024)
Graphene oxide–silica aerogel	Conductive scaffold	Electrical microenvironment support	Nerve regeneration	Preclinical (*in vitro*)	(Chen et al., 2024)
MMP-responsive mesoporous silicon	Enzyme-responsive drug delivery	Peptide cleavage in tumor microenvironment	Targeted pancreatic cancer therapy	Preclinical (animal)	(Yildirimer and Seifalian, 2014)
Magneto-silica composites	MRI + magnetic hyperthermia	Magnetic field temperature control + thermosensitive gating	Tumor targeting + monitoring	Preclinical (animal)	(Chen et al., 2024)
Boron-doped porous bioactive glass	Osteogenesis + antibiotic loading	Tunable degradation matching osteogenesis	Bone repair + anti-infection	Preclinical (animal)	(Qu et al., 2015)
Silaffin-inspired peptide silica	Biomimetic mineralization	Hierarchical structure at neutral pH	Patient-specific bone implants	Early research (concept)	(Abdelhamid and Pack, 2020)

### 8.2 Future application prospects and interdisciplinary integration

The future trajectory of silicon biomaterials depends on deep integration across disciplines—from materials science and immunology to data analytics and manufacturing engineering.

#### 8.2.1 Immuno-oncology [Speculative]

Silica nanoparticles engineered with STING agonists and checkpoint inhibitors could potentially convert “cold” tumors to “hot,” with early murine melanoma studies showing complete regression in 60% of models (Janjua et al., 2023). However, translation to human tumors—which exhibit far greater heterogeneity and immune evasion mechanisms—remains unproven. This application is currently at an early preclinical stage and faces significant challenges in dose optimization, combination scheduling, and patient stratification.

#### 8.2.2 Neurodegenerative diseases [Hypothetical]

Ultrasmall (<10 nm) silica dots that can cross the blood-brain barrier could theoretically deliver CRISPR-Cas9 components for gene editing, leveraging silicon’s renal clearance advantage over polymeric vectors (Merkel and Kissel, 2014). This concept faces formidable challenges: the efficiency of blood-brain barrier penetration by silica dots remains poorly characterized in non-human primates, CRISPR delivery efficiency and off-target editing rates require substantial improvement, and the long-term consequences of genome editing in the CNS are unknown. This application should be regarded as a long-term research aspiration rather than a near-term clinical possibility.

#### 8.2.3 AI-driven manufacturing [Emerging but unvalidated]

Digital twins of bioreactors, fed by real-time sensor data (pH, turbidity, Raman spectra), could auto-correct synthesis deviations via feedback loops, ensuring batch consistency (Chen et al., 2024). While conceptually appealing, this approach has not yet been demonstrated for pSiNP synthesis specifically. The volume and quality of sensor data required to train reliable models, the interpretability of AI-driven decisions for regulatory approval, and the cost of implementing real-time monitoring infrastructure are significant barriers.

#### 8.2.4 Blockchain-enabled supply chains [Speculative]

Blockchain systems could theoretically track recycled silicon from e-waste collection to final product, providing auditable sustainability credentials (Chen et al., 2024). However, this concept remains unproven; the added complexity and cost of blockchain infrastructure must be justified against the relatively low cost of silicon feedstock. No such system has been implemented for any nanomaterial-based medical product to date as shown in Table 8.

**TABLE 8:** Interdisciplinary innovation in manufacturing and sustainability for pSiNP development.

Innovation direction	Technical means	Core function/Goal	Supporting technologies
Intelligent Manufacturing	Digital twins of bioreactors	Auto-correct synthesis deviations, ensure batch consistency	Real-time sensor data (pH, turbidity, Raman spectra) + AI-driven feedback loops (Curtis et al., 2021)
Sustainable Development (Circular Economy)	Blockchain-enabled supply chains	Track recycled silicon from e-waste to final product, provide auditable sustainability credentials	E-waste collection + blockchain tracing (Curtis et al., 2021)

## 9 Conclusion

The research on porous silicon nanoparticles (pSiNPs) in the biomedical field has established their potential as a multifunctional, biodegradable platform that bridges diagnostics, targeted therapy, and regenerative medicine. Their tunable nanostructure, high surface area, and intrinsic biocompatibility enable precise control over drug loading, release kinetics, and cellular interactions, while degradation into orthosilicic acid minimizes long-term toxicity.

Preclinical studies have demonstrated pSiNP efficacy in non-load-bearing bone repair through silicon ion-mediated osteogenesis (2.1–2.3-fold BV/TV increase in rodent models), chronic wound healing via immunomodulation and angiogenesis (95% closure in diabetic mice), and tumor-targeted delivery leveraging passive and active homing mechanisms (up to 7-fold reduced off-target toxicity). Their utility extends to implant surface engineering and biosensing, where photoluminescence and refractive index sensitivity facilitate real-time monitoring and enhanced tissue integration.

However, it must be clearly stated that all current evidence for pSiNP biomedical applications remains at the *in vitro* or preclinical animal model stage.

Clinical translation is impeded by several unresolved challenges: the difficulty of scalable manufacturing with batch-to-batch reproducibility, unpredictable *in vivo* degradation kinetics that complicate pharmacokinetic modeling, mechanical fragility that limits load-bearing applications, production costs that exceed conventional alternatives, and the absence of harmonized characterization standards across global regulatory bodies. No pSiNP-based product has entered clinical trials as of 2026.

Emerging strategies—microfluidic synthesis for consistent production, modular surface functionalization for “plug-and-play” theranostics, and predictive in vitro–in vivo correlation models—offer potential pathways forward. Future success will depend on interdisciplinary collaboration that aligns materials innovation with defined clinical needs, adopts Quality-by-Design principles to satisfy evolving regulatory expectations, and prioritizes rigorous long-term safety evaluation. Only through systematic resolution of these translational gaps can pSiNPs advance from promising preclinical platforms toward meaningful clinical evaluation in oncology, orthopedics, dermatology, and related fields.
